# Physics-Informed Neural Network for Modeling the Pulmonary Artery Blood Pressure from Magnetic Resonance Images: A Reduced-Order Navier–Stokes Model

**DOI:** 10.3390/biomedicines13092058

**Published:** 2025-08-23

**Authors:** Sebastián Jara, Julio Sotelo, David Ortiz-Puerta, Pablo A. Estévez, Sergio Uribe, Steren Chabert, Rodrigo Salas

**Affiliations:** 1 Departamento de Informática, Universidad Técnica Federico Santa María, Santiago 8940897, Chile; jarac.sebastian@gmail.com; 2 Millennium Institute for Intelligent Healthcare Engineering (iHealth), Santiago 7820436, Chile; david.ortiz@uv.cl (D.O.-P.); pestevez@cec.uchile.cl (P.A.E.); 3 Biomedical Engineering School, Faculty of Engineering, Universidad de Valparaíso, Valparaiso 2362905, Chile; 4 Center of Interdisciplinary Biomedical and Engineering Research for Health—MEDING, Universidad de Valparaíso, Valparaiso 2540064, Chile; 5 Department of Electrical Engineering, Faculty of Physical and Mathematical Sciences, Universidad de Chile, Santiago 8370451, Chile; 6 Department of Medical Imaging and Radiation Sciences, Monash University, Melbourne, VIC 3800, Australia; sergio.uribe@monash.edu

**Keywords:** Physics-Informed Neural Network (PINN), pulmonary arterial pressure, deep learning, non-invasive techniques, cardiovascular diseases

## Abstract

**Background:** Pulmonary arterial pressure is a key parameter for diagnosing cardiovascular and pulmonary diseases. Its measurement through right heart catheterization is considered the gold standard, and it is an invasive procedure that entails significant risks for patients. This has motivated the development of non-invasive techniques based on patient-specific imaging, such as Physics-Informed Neural Networks (PINNs), which integrate clinical measurements with physical models, such as the 1D reduced Navier–Stokes model, enabling biologically plausible predictions with limited data. **Methods:** This work implements a PINN model that uses velocity and area measurements in the main bifurcation of the pulmonary artery, comprising the main artery and its secondary branches, to predict pressure, velocity, and area variations throughout the bifurcation. The model training includes penalties to satisfy the laws of flow and momentum conservation. **Results:** The results show that, using 4D Flow MRI images from a healthy patient as clinical data, the pressure estimates provided by the model are consistent with the expected ranges reported in the literature, reaching a mean arterial pressure of 21.5 mmHg. **Conclusions:** This model presents an innovative approach that avoids invasive methods, being the first study to apply PINNs to estimate pulmonary arterial pressure in bifurcations. In future work, we aim to validate the model in larger populations and confirm pulmonary hypertension cases diagnosed through catheterization.

## 1. Introduction

Computational fluid dynamics (CFD) has become a key tool for in silico analysis of the cardiovascular system, enabling applications in medical device design, surgical planning, diagnostics, and non-invasive disease assessment [1]. In particular, cardiovascular flow modeling provides valuable insight into complex hemodynamic behavior, aiding physiological understanding and clinical decision making [2]. Within this context, pulmonary arterial pressure (PAP) is a key biomarker for evaluating cardiopulmonary health and detecting conditions such as pulmonary hypertension. The gold standard for PAP measurement is right-heart catheterization, an invasive and often costly procedure associated with risks that include infection and vascular injury [3,4,5,6]. This has driven the development of non-invasive alternatives such as Doppler ultrasound and optical coherence tomography, which offer important information on blood flow dynamics. However, these methods are typically limited to specific anatomical regions and may suffer from noise, low spatial resolution, or indirect estimations of pressure [7]. As a result, there is a growing need for complementary, non-invasive tools that enable the reliable estimation of pressure fields and support the early study of disease onset and progression.

Physics-informed neural networks (PINNs) have emerged as a powerful framework for modeling dynamical systems governed by physical laws [8]. By incorporating partial differential equations—such as the reduced-order (1D) formulations of the Navier–Stokes equations—directly into the learning process, these networks can estimate key variables with a reduced reliance on experimental data [9]. This enables the reconstruction of unobserved hemodynamic quantities, such as blood flow and pressure [10], even from limited and noisy measurements [11,12], making PINNs particularly attractive for medical applications, including the assessment of pulmonary arterial pressure. A pioneering study by Georgios Kissas et al. in 2020 demonstrated the feasibility of using PINNs to estimate pressure, velocity, and cross-sectional area in human arteries from sparse clinical data [13]. Building on this, Mohammad Sarabian et al. (2021) applied a similar strategy to the diagnosis of cerebral vasospasm, confirming the potential of PINNs to integrate clinical observations with physical models in complex physiological settings [14]. More recently, Li et al. introduced a novel PINN-based framework for cuffless blood pressure estimation, combining physiological signals with 1D Navier–Stokes equations to reconstruct pressure waveforms from non-invasive data [15]. In parallel, Csala et al. proposed a physics-constrained neural differential equation approach that enhances the accuracy and computational efficiency of 1D cardiovascular models, showing robustness to noise and uncertainty in boundary conditions [16]. Collectively, these contributions establish a strong foundation for the non-invasive estimation of pulmonary artery pressure using PINNs combined with pulsatile 1D Navier–Stokes flow models.

Complementary to physics-informed approaches, purely data-driven methods have also been explored for non-invasive estimation of pulmonary artery pressure (PAP). These rely on machine learning models trained on clinical, imaging, or physiological data—such as ECG, chest X-rays, Doppler echocardiography, or cardiac MRI—to identify patterns associated with pulmonary hypertension. Recent studies have reported high classification accuracy for elevated PAP using deep learning applied to ECG and radiographs [17,18], as well as strong agreement with catheterization-based pressure estimates using cine MRI [19]. Multimodal echocardiographic frameworks have also improved over traditional Doppler-based methods [20], while wearable technologies like photoplethysmography and digital auscultation show promise for large-scale, non-invasive screening [21]. These models offer flexible, data-centric alternatives in scenarios where invasive techniques or high-resolution imaging are not feasible.

A notable limitation in the application of 1D Navier–Stokes models is the challenge of estimating patient-specific mechanical parameters—such as arterial wall stiffness—directly from imaging data. These parameters, which strongly influence pressure and flow predictions, are often approximated from literature values or geometric surrogates, potentially limiting the model’s accuracy and clinical applicability [22,23]. Additionally, 1D formulations inherently neglect complex three-dimensional flow phenomena, such as turbulence, secondary flows, and vortices at bifurcations or regions with abrupt geometric changes. These simplifications may lead to local discrepancies compared to full 3D computational fluid dynamics approaches.

Despite these limitations, 1D models remain highly valuable in hemodynamic research and clinical applications. Their computational efficiency allows for rapid simulations of flow and pressure across extensive vascular networks with limited computational resources, making them particularly attractive for patient-specific studies, population screening, and scenarios where local 3D flow features are of secondary importance [24,25]. The reduced-order nature of 1D models also facilitates integration with data-driven frameworks, such as PINNs, enabling fast and non-invasive pressure estimation from medical imaging data, as demonstrated in this study.

This work presents a PINN-based model as a proof of concept for the non-invasive estimation of pulmonary artery pressure using limited clinical data on velocity and cross-sectional area. Our approach employs a one-dimensional representation of arterial hemodynamics based on the ROM Navier–Stokes equations, incorporating continuity conditions at the Y-shaped bifurcation of the main arteries. The proposed framework builds upon the methodology of Kissas et al. [13] but is specifically applied to the pulmonary artery. The model was validated using publicly available numerically simulated data, ensuring its robustness before applying it to real clinical cases. A key innovation of this study is the estimation of mechanical parameters directly from blood flow data obtained via 4D Flow MRI. The estimated pressure values align well with reported physiological ranges, demonstrating the feasibility of this approach for non-invasive hemodynamic assessment.

## 2. Methods

### 2.1. Reduced-Order Flow Model for the Pulmonary Artery

For this work, we adopt the hemodynamic assumptions and the mathematical model proposed by [13]. In this approach, the ROM model (1D) assumes that the arterial curvature is small enough to describe the geometry by Cartesian coordinates. In addition, the fluid is considered incompressible and Newtonian, with constant density and viscosity. The structural properties of the artery are preserved at each cross-section, and the system is completed by an equation linking pressure to the area, complementing the equations of conservation of mass and quantity of motion. Furthermore, the model assumes that the vessel is a straight cylinder oriented along the *z*-axis, with wall displacements occurring only in the radial direction, ensuring circular cross-sections where blood pressure and flow rate remain constant, while all body forces, including gravity, are neglected [15]. Using the ROM Navier–Stokes equations, a 1D model for blood flow in a non-rigid artery is derived to describe hemodynamics in the pulmonary artery:

(1) $$\frac{\partial A}{\partial t}+A\frac{\partial u}{\partial x}+u\frac{\partial A}{\partial x}=0,$$

(2) $$\frac{\partial u}{\partial t}+\alpha u\frac{\partial u}{\partial x}+\frac{u}{A}\frac{\partial (\alpha -1)uA}{\partial x}+\frac{1}{\rho }\frac{\partial p}{\partial x}-K_{R}\frac{u}{A}=0,$$

(3) $$p-p_{\mathrm{ext}}-\beta \left(\sqrt{A}-\sqrt{A_{0}}\right)=0,$$

where A(x,t), u(x,t), and p(x,t) represent the cross-sectional area, velocity, and pressure, respectively; β is the mechanical parameter in the constitutive law relating pressure and area (see Equation (3)) and is defined as $\beta =\frac{\sqrt{\pi }h_{0}E}{\left(1-\nu^{2}\right)A_{0}}$. Here, $A_{0}$, $h_{0}$, *E*, ν, and $p_{ext}$ denote the equilibrium cross-sectional area, wall thickness, Young’s modulus, Poisson ratio, and external pressure of the artery, respectively. The value of β is estimated according to the procedure later described in Section 2.3. ρ, α, and $K_{R}$ represent the blood density, the momentum flux correction factor, and the friction parameter, respectively.

Each of the equations in the system plays a key role in capturing the essential physics of pulmonary hemodynamics. Equation (1) enforces conservation of mass, ensuring the continuity of flow along the vessel. Equation (2) corresponds to the momentum balance, accounting for convective acceleration, pressure gradients, and viscous losses through the term involving $K_{R}$. Finally, Equation (3) establishes a nonlinear constitutive relation between pressure and area, which reflects the elastic deformation of the arterial wall under pulsatile loading. Together, these equations govern the spatiotemporal evolution of pressure and flow in the pulmonary artery and enable the inference of pressure fields from area and velocity measurements.

The one-dimensional model described above can be extended to address arterial bifurcations by applying appropriate conditions to the pressure, velocity, and area variables at the bifurcation point. This work focuses on the main artery and its daughter branches, representing the main artery and its first bifurcation. The conservation of total flow states that the flow $Q_{1}$ at the end of the main artery must equal the sum of the flows $Q_{2}$ and $Q_{3}$ in the daughter arteries at the initial points of their respective centerlines. To establish this condition, the conservation of linear momentum transported by the fluid through the shared region is applied under the assumption of no losses, vortex formation, or separation. Mathematically, this relationship is expressed as

(4) $$A_{1}\;u_{1}=A_{2}\;u_{2}+A_{3}\;u_{3},$$

(5) $$p_{1}+\frac{\rho \;u_{1}^{2}}{2}=p_{2}+\frac{\rho \;u_{2}^{2}}{2}=p_{3}+\frac{\rho \;u_{3}^{2}}{2}.$$

### 2.2. Physics-Informed Neural Networks

**Figure 1:**
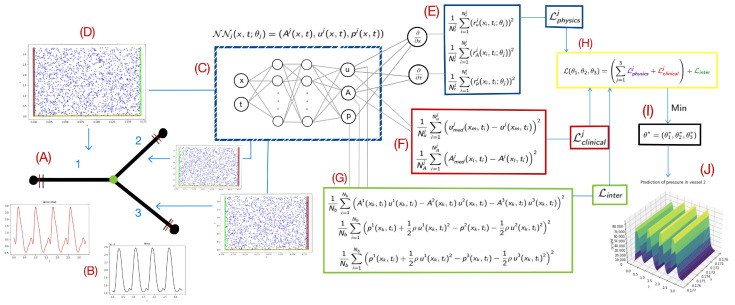
Methodology framework implemented: In (**A**), a simplified representation of the bifurcation is illustrated, while in (**B**), the clinical measurements of area and velocity are incorporated at the inlet and outlet of the bifurcation. The inputs and outputs of the ANNs are detailed in (**C**). In (**D**), the domains of each network are depicted, highlighting collocation points (blue), interface points (green), and the locations of velocity (red) and area (black) measurements. The loss functions are broken down in (**E**) for collocation points, (**F**) for clinical measurements, (**G**) for continuity at the bifurcation, and (**H**) for the total loss composition. Finally, (**I**,**J**) depict the arterial pressure estimation.

Following the approach of [8], we consider the problem where we have partial information on the unknown variables A(x,t),u(x,t), i.e., cross-sectional area and velocity, and completely missing information about the pressure, p(x,t), at a time instant *t* and a position *x*. For each vessel *j*, the objective is to obtain an approximation of the unknown functions using a feedforward fully connected neural network (ANN) that we denote as ${\mathrm{NN}}_{j}\left(x,t;{\vec{\theta }}_{j}\right)$, i.e.,

(6) $$\left(A^{j}\left(x,t\right),u^{j}\left(x,t\right),p^{j}\left(x,t\right)\right)\approx \left({\hat{A}}^{j}\left(x,t;{\vec{\theta }}_{j}\right),{\hat{u}}^{j}\left(x,t;{\vec{\theta }}_{j}\right),{\hat{p}}^{j}\left(x,t;{\vec{\theta }}_{j}\right)\right):={\mathrm{NN}}_{j}\left(x,t;{\vec{\theta }}_{j}\right),$$

where ${\hat{A}}^{j}$, ${\hat{u}}^{j}$, and ${\hat{p}}^{j}$ are the ANN output approximations of the area, velocity, and pressure in artery *j*, and ${\vec{\theta }}_{j}$ are the trainable parameters of each j − ANN. To train each neural network, we define a composite loss function comprising three components: (i) a physics-based loss term enforcing the residuals of the governing 1D Navier–Stokes Equations (1)–(3), which ensures that predictions remain consistent with mass and momentum conservation; (ii) a data loss term that penalizes discrepancies between model predictions and available imaging measurements; and (iii) an interface loss that enforces the conservation of linear momentum (4) and (5). To achieve this approximation, we define the total cost function as

(7) $$L\left({\vec{\theta }}_{1},{\vec{\theta }}_{2},{\vec{\theta }}_{3}\right):=\left(\sum_{j=1}^{3}L_{NS}^{j}+L_{data}^{j}\right)+L_{inter},$$

where the cost functions $L_{NS}^{j}$ and $L_{data}^{j}$ correspond to the physical model and clinical data, respectively, for the three segments j=1,2,3, and $L_{inter}$ represents the cost function associated with the interface connecting the three segments. For each *j*-th ANN, the cost function $L_{NS}^{j}$ is defined as

(8) $$L_{NS}^{j}:=\frac{1}{N_{r}^{j}}\sum_{i=1}^{N_{r}^{j}}{\left(r_{A}^{j}\left({\hat{x}}_{i}^{*},{\hat{t}}_{i}^{*};\theta_{j}\right)\right)}^{2}+\frac{1}{N_{r}^{j}}\sum_{i=1}^{N_{r}^{j}}{\left(r_{u}^{j}\left({\hat{x}}_{i}^{*},{\hat{t}}_{i}^{*};\theta_{j}\right)\right)}^{2}+\frac{1}{N_{r}^{j}}\sum_{i=1}^{N_{r}^{j}}{\left(r_{p}^{j}\left({\hat{x}}_{i}^{*},{\hat{t}}_{i}^{*};\theta_{j}\right)\right)}^{2},$$

where $r_{A}$, $r_{u}$, and $r_{p}$ are the dimensionless and normalized residuals of (1), (2), and (3) respectively, i.e.,

$$\begin{array}{ccc} r_{A}^{j}\left({\hat{x}}^{*},{\hat{t}}^{*};\theta_{j}\right) & := & \frac{1}{\sigma_{t^{*}}^{j}}\frac{\partial {\hat{A}}^{*j}}{\partial {\hat{t}}^{*}}+\frac{1}{\sigma_{x*}^{j}}{\hat{A}}^{*j}\frac{\partial {\hat{u}}^{*j}}{\partial {\hat{x}}^{*j}}+\frac{1}{\sigma_{x*}^{j}}{\hat{u}}^{*j}\frac{\partial {\hat{A}}^{*j}}{\partial {\hat{x}}^{*j}},\\ r_{u}^{j}\left({\hat{x}}^{*},{\hat{t}}^{*};\theta^{j}\right) & := & \frac{1}{\sigma_{t*}^{j}}\frac{\partial {\hat{u}}^{*j}}{\partial {\hat{t}}^{*j}}+\frac{1}{\sigma_{x*}^{j}}\alpha \;{\hat{u}}^{*j}\frac{\partial {\hat{u}}^{*j}}{\partial {\hat{x}}^{*j}}+\frac{1}{\sigma_{x*}^{j}}{\hat{u}}^{*j}\frac{\partial \left(\alpha -1\right){\hat{u}}^{*j}{\hat{A}}^{*j}}{\partial {\hat{x}}^{*j}}+\frac{1}{\sigma_{x*}^{j}}\frac{\partial {\hat{p}}^{*j}}{\partial {\hat{x}}^{*j}}-\frac{K_{R}}{LU}\frac{{\hat{u}}^{*j}}{{\hat{A}}^{*j}},\\ r_{p}^{j}\left({\hat{x}}^{*},{\hat{t}}^{*};\theta^{j}\right) & := & {\hat{p}}^{*j}\;p_{0}-p_{ext}-\beta \left(\sqrt{{\hat{A}}^{*j}A^{0}}-\sqrt{A_{0}}\right), \end{array}$$

with ${\hat{A}}^{*}$, ${\hat{u}}^{*}$, and ${\hat{p}}^{*}$ are the dimensionless unknowns; ${\hat{t}}^{*}$ and ${\hat{x}}^{*}$ are the dimensionless and normalized spatial–temporal domain; and $\sigma_{t^{*}}$ and $\sigma_{x^{*}}$ are the standard deviations of the spatial–temporal domain. See Appendix A for further details about variables’ non-dimensionalization, normalization, and recovery. In (8), $x_{i}$ and $t_{i}$ represent the blue collocation points within the network domain, as shown in Figure 1D. In this case, if the cost function approaches zero, the ANN satisfies the differential equations of system (1) to (3). On the other hand, for each *j*-th ANN, the data loss is defined as follows:

(9) $$L_{data}^{j}:=\frac{1}{N_{u}^{j}}\sum_{i=1}^{N_{u}^{j}}{\left({u^{*}}_{x_{m},t_{i}}^{j}-{\hat{u}}^{*j}\left(x_{m},t_{i};\theta_{j}\right)\right)}^{2}+\frac{1}{N_{A}^{j}}\sum_{i=1}^{N_{A}^{j}}{\left({A^{*}}_{x_{l},t_{i}}^{j}-{\hat{A}}^{*j}\left(x_{l},t_{i};\theta_{j}\right)\right)}^{2}.$$

Notice that in (9), the first term on the right-hand side aligns the network’s predictions with the observed velocity data at fixed locations $x_{m}$ (represented by the boundary red points in Figure 1D). In contrast, the second term ensures agreement with observed area data at locations $x_{l}$ (boundary black points). For each vessel, both velocity and area measurements from the clinical data are imposed at the ends of each segment and can be interpreted as Dirichlet boundary conditions for *u* and *A*. These constraints provide the necessary physical information to ensure the well-posedness and convergence of the PINN solution. Notably, pressure is not imposed at the boundaries, as it is the variable to be estimated by the model. Finally, the interface loss is defined from (4) and (5) as follows:

(10) $$\begin{array}{cc} L_{inter}:= & \frac{1}{N_{b}}\sum_{i=1}^{N_{b}}{\left({\hat{A}}_{1}^{*}\left(x_{k},t_{i};\theta_{1}\right){\hat{u}}_{1}^{*}\left(x_{k},t_{i};\theta_{1}\right)-{\hat{A}}_{2}^{*}\left(x_{k},t_{i};\theta_{2}\right){\hat{u}}_{2}^{*}\left(x_{k},t_{i};\theta_{2}\right)-{\hat{A}}_{3}^{*}\left(x_{k},t_{i};\theta_{3}\right){\hat{u}}_{3}^{*}\left(x_{k},t_{i};\theta_{3}\right)\right)}^{2}\\ + & \frac{1}{N_{b}}\sum_{i=1}^{N_{b}}{\left({\hat{p}}_{1}^{*}\left(x_{k},t_{i};\theta_{1}\right)+\frac{1}{2}\rho {\left({\hat{u}}_{1}^{*}\right)}^{2}\left(x_{k},t_{i};\theta_{1}\right)-{\hat{p}}_{2}^{*}\left(x_{k},t_{i};\theta_{2}\right)-\frac{1}{2}\rho {\left({\hat{u}}_{2}^{*}\right)}^{2}\left(x_{k},t_{i};\theta_{2}\right)\right)}^{2}\\ + & \frac{1}{N_{b}}\sum_{i=1}^{N_{b}}{\left({\hat{p}}_{1}^{*}\left(x_{k},t_{i};\theta_{1}\right)+\frac{1}{2}\rho \;{\left({\hat{u}}_{1}^{*}\right)}^{2}\left(x_{k},t_{i};\theta_{1}\right)-{\hat{p}}_{3}^{*}\left(x_{k},t_{i};\theta_{3}\right)-\frac{1}{2}\rho \;{\left({\hat{u}}_{3}^{*}\right)}^{2}\left(x_{k},t_{i};\theta_{3}\right)\right)}^{2}, \end{array}$$

where $x_{k}$ are the collocation points over the interface. Notice that the number and locations of the points must be the same for the three ANNs. Figure 1 summarizes the approach to model the arterial bifurcation.

All tests were performed using an ANN architecture consisting of 2 input neurons for the temporal and spatial variables; 7 hidden layers with 100 neurons each, with hyperbolic tangent as activation function; and 3 output neurons for the area, pressure, and flow for each vessel *j*. The training was performed using the ADAM optimizer. Also, we used a Glorot initialization scheme for the ANNs’ trainable parameters [26]. All codes were implemented in Python 3.11 with TensorFlow 2.11 [27]. The computations were executed on a MacBook Pro with an M1 chip (8 cores). The full implementation, including all the codes used for this study, is available at https://github.com/sevaseba/PINN-presion-bifurcacion-arterial.git (accessed on 14 August 2025).

### 2.3. Implementation in a Clinical Case

Before implementation with clinical data, we performed a validation of the proposed method using public, synthetic data generated by Kissas et al. using simulators based on the Discontinuous Galerkin method to solve (1) to (3). These data correspond to the area, blood velocity, and pressure in arteries with Y-shaped bifurcation and allowed us to obtain the mean square error with respect to these reference solutions. The objective of this validation was to verify the consistency between the results obtained with our implemented code and those previously reported in the literature. Full details of the validation process are available in the Appendix B.

**Figure 2:**
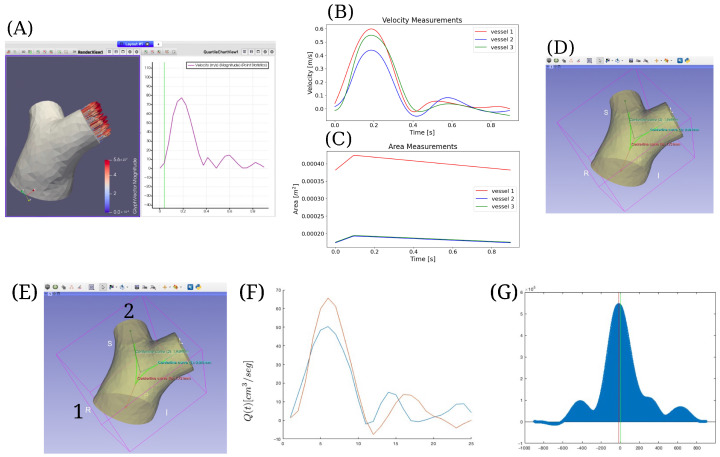
(**A**) Processing in ParaView. (**B**,**C**) Processing in TensorFlow. (**D**,**E**) Selection of longest section. (**F**) Average velocity curves at points 1 and 2. (**G**) Shift interval calculated using cross-correlation.

For the clinical implementation, measurements were obtained from 4D Flow MRI images of the pulmonary artery bifurcation in a healthy volunteer recruited in a previously reported study [28]. These images were processed using the 4D Flow Matlab Toolbox [29], which allowed manual segmentation of the arterial bifurcation and quantification of hemodynamic parameters such as velocity and arterial geometry. To preserve the validity of the one-dimensional flow assumption in the Navier–Stokes model, the proximal portions of both the main and secondary pulmonary arteries were slightly trimmed, removing regions with strong curvature or geometric irregularities and ensuring straighter inlet and outlet profiles before the computational extraction. The results were exported to ParaView, where the main artery and its bifurcation were extracted, along with the time series of velocity and area at the extremities of each branch; see Figure 2A.

The velocity series, consisting of 34 frames between 0 and 0.89 s, were smoothed using second-order Lagrangian interpolation, generating the curves shown in Figure 2B,C. The maximum cross-sectional area of each vessel section was directly obtained from the 4D Flow data, while the minimum area was estimated assuming a physiological variation of approximately 10% between the peak and trough values. This assumption is supported by the literature, reporting that the largest area occurs when the slope of the velocity curve reaches its maximum [30]. The derived maximum and minimum areas for each branch, as illustrated in Figure 2C, were used as boundary inputs for the computational model to represent vessel compliance in the absence of a fully non-rigid arterial wall reconstruction.

The vascular surface generated in ParaView was then imported into 3D Slicer, where the centerlines of the three main pulmonary branches were extracted using the VMTK module [31]; see Figure 2D. Vessel lengths were calculated in 3D Slicer using this method, and the final values used in the simulation are reported in Table 1 in the second column.

The values of the parameter β reported in the third column of Table 1 were computed using the β expression described in Section 2.1, which relates Young’s modulus *E* to the pulse wave velocity $c=\sqrt{\frac{Eh_{0}}{2\rho R_{0}}}$, assuming both *c* and *E* are constant. Calculating the wave velocity *c* is crucial, as it varies among patients [32]. For this purpose, the broader section of the pulmonary artery is selected, as illustrated in Figure 2E, and the distance between points 1 and 2 is computed using 3D Slicer software, where, for this case, the measured distance is 0.0621 m. Then, at points 1 and 2 of the bifurcation, the average velocity curves are obtained, as shown in Figure 2F. The velocity at which the wave propagates is measured from these curves to estimate *c*. To determine the time required for the pulse wave to travel between points 1 and 2, the time shift between the two curves in Figure 2F is calculated. To enhance accuracy, a Gaussian regression model is fitted to each velocity curve, and the time shift is determined via cross-correlation between both Gaussian models; see Figure 2G. The resulting time shift interval was 0.0155 s. With these values, the estimated pulse wave velocity was $c=\frac{0.0421\;m}{0.0155\;s}=4.01\;m/s$. Knowing *c*, a Young’s modulus of 340,898.12 Pa was obtained. Using *E*, β was then computed using the expression described in Section 2.1, considering ν=0.5 and a constant $h_{0}$ equivalent to 10% of the radius $R_{0}$ [30], where $R_{0}$ corresponds to the radius at the minimum area $A_{0}$ reported in the last column of Table 1. To simplify the model, the cross-sectional area at equilibrium $A_{0}$ was assumed constant along the vessel length, as the variations between inlet and outlet cross-sectional areas are relatively small, as reported in [33].

## 3. Results

After processing the 4D Flow image and obtaining the clinical parameters through the methodology described in Section 2.3, we trained the PINNs following the scheme depicted in Figure 1 to estimate the blood pressure at the arterial bifurcation. We used the loss function in (7), the β parameters and initial areas $A_{0}$ from Table 1, and assumed ρ=1060 kg/m^3^. In large pulmonary arteries with high Womersley numbers, the velocity profile remains nearly flat, and viscous energy losses are minimal along short, straight segments. The thin boundary layer formed in these vessels contributes negligibly to the net momentum balance, making its effect on pressure predictions minor compared to inertial and convective terms [24,33]. Therefore, we set α=1 and $K_{R}=0$ as a reasonable simplification with negligible expected impact on predicted pressure waveforms [34] while also reducing the number of uncertain parameters and computational cost of the simulations. The external pressure $p_{ext}$ in all three vessels was set to 16 mmHg, and the initial velocity condition was set to zero. A total of 2000 collocation points were randomly distributed across the spatiotemporal domain, and 101 were assigned to the initial and boundary conditions.

**Figure 3:**
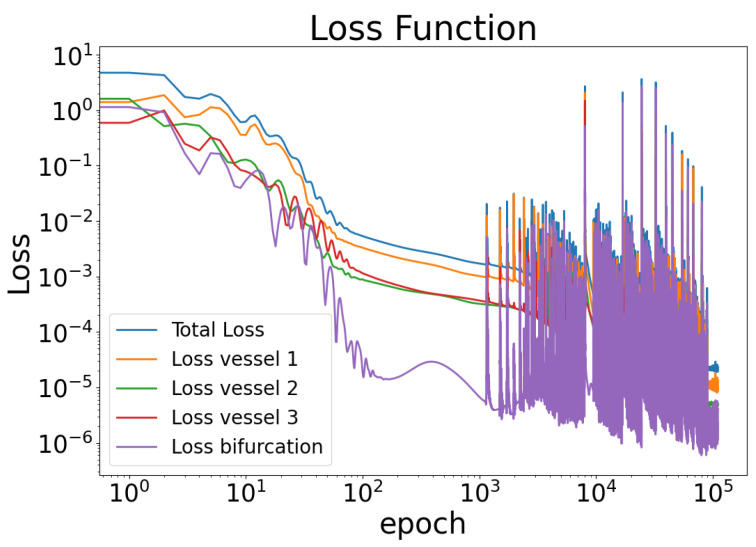
Loss function decay over 50,000 epochs during training using the Adam optimizer with a fixed learning rate of 0.001. The total loss and individual losses for each vessel and the bifurcation are shown. The plot uses a log-log scale to highlight convergence trends and relative magnitudes across components.

**Figure 4:**
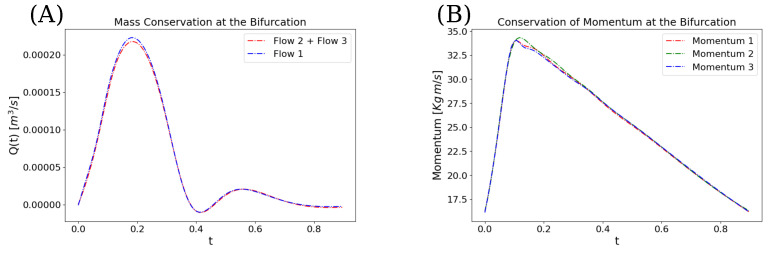
Comparison of conserved quantities at the bifurcation point after training the artificial neural networks (ANNs). (**A**) Mass conservation: the total flow at the bifurcation remains consistent with the sum of flows from vessels 2 and 3. (**B**) Momentum conservation: the momentum in all three vessels aligns, confirming the preservation of conservation laws in the proposed model.

Figure 3 shows the evolution of the loss function during training over 50,000 epochs using the Adam optimizer with a fixed learning rate of 0.001. The total loss and the individual contributions from each vessel segment and the bifurcation region are displayed, showing a consistent decrease followed by stabilization, with the bifurcation loss exhibiting the most significant reduction. Furthermore, Figure 4 demonstrates that all three networks’ continuity (4) and (5) are satisfied at the bifurcation point. It can be inferred from Figure 4 that the flow in vessel 1 equals the sum of the flows in vessels 2 and 3 at the bifurcation point. Additionally, as shown in Figure 4B, the momentum and mass conservation of all three vessels remain closely aligned at the bifurcation, suggesting that the proposed model effectively enforces the conservation laws, albeit with minor deviations at peak values.

**Figure 5:**
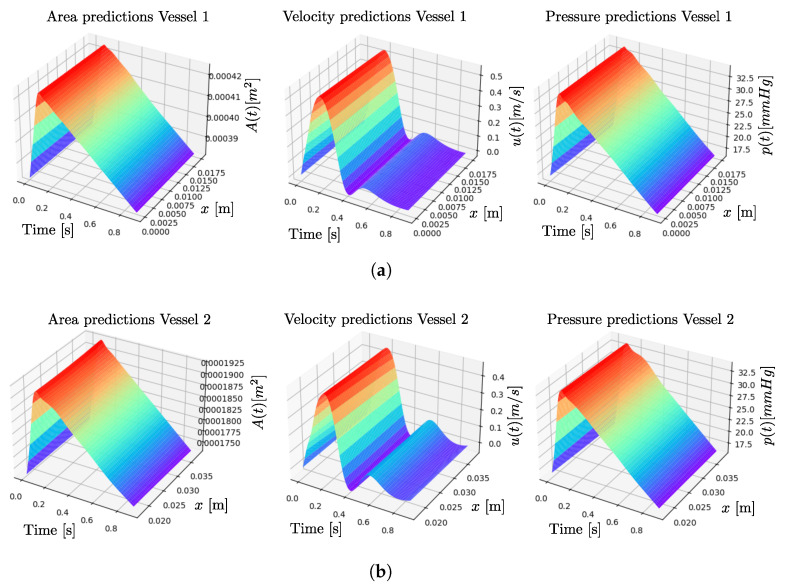

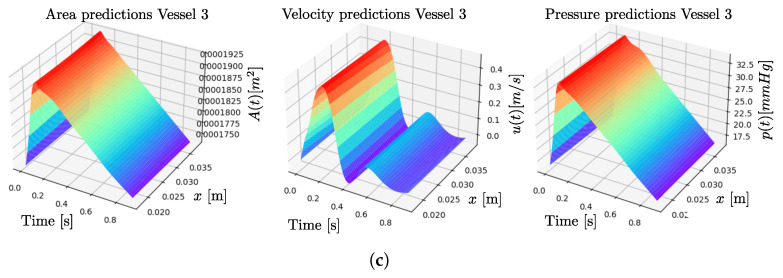
Predictions of area, velocity, and pressure along the arterial bifurcation at different spatial locations. (**a**) Results for artery 1, (**b**) results for artery 2, and (**c**) results for artery 3. Each plot displays the estimated variable at five distinct points along the corresponding artery, as indicated by the *x* values.

Figure 5 presents the simulation results as 3D surface plots. Each row corresponds to a different artery (from top to bottom: vessels 1 to 3), and each column shows the predicted cross-sectional area, velocity, and pressure (from left to right). The surfaces represent the spatiotemporal evolution of each quantity along the vessel length (*x*-axis) and over time (*t*-axis), providing a comprehensive view of the dynamic hemodynamic behavior captured by the model. The predicted pressure values range approximately from 16 mmHg during early and late diastole to 32.5 mmHg at peak systole. Using the standard formula for mean pulmonary arterial pressure, $\mathrm{mPA}=\frac{2P_{d}+P_{s}}{3}$, a mean value of 21.5 mmHg was obtained, which aligns with the expected range for healthy individuals reported in [5,35].

## 4. Discussion

This study developed and implemented a physics-informed neural network (PINN) methodology to estimate pulmonary arterial pressure non-invasively at arterial bifurcations using 4D Flow MRI images. Applying the method to real clinical data resulted in physiologically plausible pressure estimations for a healthy subject, with predicted values around 21.5 mmHg, consistent with ranges reported in the literature for normal pulmonary hemodynamics [5,35]. This outcome suggests that, by leveraging routinely available imaging data and combining them with a reduced-order physical model, it is possible to estimate pressure fields in the pulmonary artery without the need for invasive catheterization. To our knowledge, no previous study has proposed a PINN framework to estimate pressure specifically at the pulmonary artery bifurcation from non-invasive data, making this a novel contribution to the field.

Our proposed methodology allowed us to estimate a patient-specific value of the arterial wall Young’s modulus *E* directly from 4D Flow MRI data, yielding a value of approximately 3.4 kPa. This estimate lies within the range of 5.44 ± 2.96 kPa reported for pulmonary arterial tissue in healthy human samples [36], supporting the physiological plausibility of the approach. Accurate estimation of vessel wall stiffness, captured by the parameter β, is essential, as small deviations in its value can lead to substantial errors in the reconstructed pressure field [32]. Since β is related to the nonlinear constitutive law in Equation (3), an over- or underestimation may result in erroneous pressure predictions or even yield pathological results in otherwise healthy cases [34]. The strategy we propose, which relies on pulse wave velocity measurements derived from non-invasive imaging, provides a viable means of incorporating patient-specific mechanical properties into the PINN framework, reducing reliance on literature-based surrogates and potentially enhancing the fidelity of hemodynamic predictions [23]. However, further work is needed to better understand the potential sources of error in estimating this parameter with the proposed method.

One-dimensional (1D) models offer important advantages for arterial hemodynamics simulations, particularly their computational efficiency and ability to scale to large vascular networks [24,25]. These properties make them well suited for non-invasive pressure estimation from imaging data, as demonstrated in this work. Our methodology combined 4D Flow MRI with a 1D reduced-order model, allowing patient-specific predictions of flow and pressure with low computational cost. However, 1D models inherently neglect complex three-dimensional flow features, such as turbulence, secondary flows, and vortices at bifurcations, which may lead to local discrepancies compared to full 3D CFD approaches [22].

In our PINN framework, the physical laws are incorporated as soft constraints via penalty terms in the loss function rather than as strict algebraic equalities at every point [8,37]. Consequently, the network is trained to minimize the residuals associated with these equations at the collocation points, aiming for—but not guaranteeing—exact conservation throughout the domain. This weak enforcement can be observed in Figure 4, which shows the conservation of mass and momentum at the bifurcation point. Although small differences are present between the three vessels in both quantities, the maximum discrepancy in mass conservation remains minor. Nevertheless, the model successfully predicts a physiologically plausible pressure of 21.5 mmHg in the pulmonary artery, which aligns with expected values from the literature [30].

The present study has certain limitations that should be addressed in future contributions. The lack of direct in vivo arterial pressure measurements restricts validation to indirect comparisons with reported values and methods from similar studies, highlighting the need for catheter-based validation [13,14]. Additionally, the dataset is limited, making this more of a proof of concept than a full clinical validation, and its applicability to pathological cases like pulmonary hypertension remains unexplored. To advance toward clinical translation, future work should include a broader patient cohort encompassing both healthy individuals and patients with confirmed pathology by RHC. Another limitation relates to the uncertainty in key input parameters, such as vessel lengths and the stiffness parameter β. Although these parameters were estimated from image-derived measurements, no formal sensitivity analysis was performed to quantify their potential impact on model predictions. Variations in these parameters could influence the estimated pressure field and overall model robustness, particularly in patient-specific applications where measurement noise or segmentation errors are present. In addition, the assumption of a zero friction coefficient ($K_{R}=0$) in the 1D momentum equation neglects linear viscous energy losses along the vessel, which may result in a slight underestimation of pressure drops in more realistic scenarios where wall shear effects are not entirely negligible [24]. Future work should revisit these assumptions through sensitivity analyses to assess their impact on predicted pressure fields. Extending the model to more complex vascular topologies could enhance its clinical utility. Future work includes expanding the dataset, performing ablation studies on ANN hyperparameters and loss terms, testing alternative ANN models, incorporating more arterial branches and measurement sections, refining boundary conditions with Windkessel parameters, improving patient-specific estimation of the β value, and conducting sensitivity analyses to assess the influence of parameter variability on the predictive accuracy and stability of the proposed framework. These steps will enhance the model’s predictive accuracy and clinical relevance, advancing non-invasive pulmonary hemodynamics assessment.

## 5. Conclusions

We developed a PINN-based framework combining a 1D Navier–Stokes model with 4D Flow MRI data to estimate pulmonary arterial pressure at arterial bifurcations non-invasively. The method produced physiologically consistent results in a healthy subject and enabled patient-specific estimation of vessel stiffness from imaging data. To our knowledge, this is the first study to apply PINNs for pressure estimation specifically at the pulmonary artery bifurcation from non-invasive measurements. Future work will focus on extending the approach to larger datasets, refining model parameters, evaluating performance in pathological cases, and improving robustness through sensitivity analyses and advanced network architectures.

## Figures and Tables

**Table 1:** Physiological parameters used in the simulation estimated according to the procedure described in Section 2.3.

Artery Segment	Length (m)	β (Pa/m)	Cross-Sectional Area at Equil. (m^2^)
1	0.01763	$2.32586\times 10^{6}$	$3.8191\times 10^{-4}$
2	0.01892	$3.45194\times 10^{6}$	$1.7338\times 10^{-6}$
3	0.02024	$3.43681\times 10^{6}$	$1.7491\times 10^{-5}$

**Figure A1:**
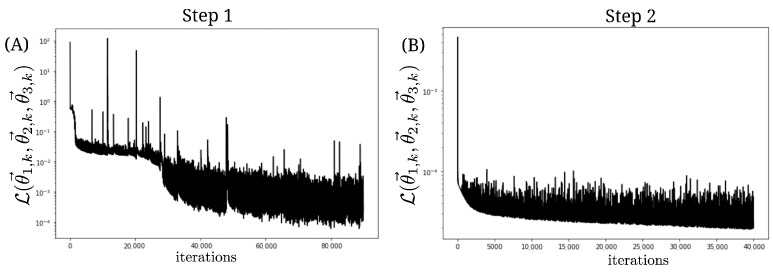
Loss function values versus the number of iterations of the Adam algorithm during training. (**A**) First 90,000 iterations with a learning rate of 0.001. (**B**) Final 40,000 iterations with a learning rate of 0.0001.

**Figure A2:**
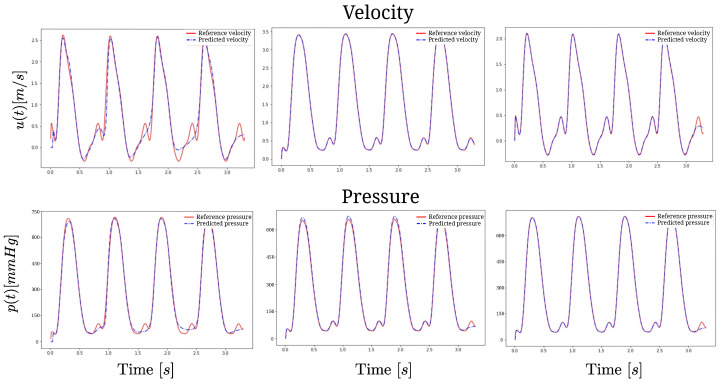
Comparison between the PINN model and the Discontinuous Galerkin reference solution.

## Data Availability

The data used in this study can be obtained from their original repositories: https://pubmed.ncbi.nlm.nih.gov/19672940/ (accessed on 19 August 2025), and 1DBloodFlowPINNs from https://github.com/PredictiveIntelligenceLab/1DBloodFlowPINNs (accessed on 19 August 2025).

## Abbreviations

The following abbreviations are used in this manuscript:

PINNs	Physics-Informed Neural Networks
MRI	Magnetic Resonance Image
ANN	Artificial Neural Network
DG	Discontinuous Galerkin

## Appendix A. Variables Non-Dimensionalization and Normalization

Before computational implementation, it is important to note that the orders of magnitude of the physical quantities in the system of Equations (1) to (3) differ significantly. For example, these include $p∼10^{6}$ Pa, $A∼10^{-5}$ m^2^, and u∼10 m/s. This difference complicates the training of neural networks, since such scale variations severely impact the magnitude of the gradients of the loss function L during the backpropagation algorithm, which adjusts the neural network weights [26]. To address this issue, a non-dimensionalization process is applied to scale the inputs and outputs of the neural networks, specifically ensuring that $\left({\hat{A}}^{*},{\hat{u}}^{*},{\hat{p}}^{*},x^{*},t^{*}\right)∼O\left(1\right)$. Additionally, a normalization process is performed on the spatial and temporal coordinates so that they have zero mean and unit variance, which is a standard practice in deep learning. Following Kissas et al. [13], the characteristic length *L* and velocity *U* are defined as

(A1) $$L:=\sqrt{\frac{1}{3}\sum_{j=1}^{3}A_{0}^{j}}=0.01156\;m,\;U:=10\;m/s,$$

where *L* is the square root of the average cross-sectional areas $A_{0}^{j}$ of each vessel *j* presented in Table 1. The choice of U=10 m/s is based on the physiological observation that in large arteries, the wave speed is approximately one order of magnitude greater than the vessel length [13]. These characteristic quantities are used to scale the spatial and temporal domains, which helps mitigate numerical instabilities during training by reducing the magnitude discrepancy between variables, thereby improving gradient-based optimization. Thus, the inputs x,t and outputs $\hat{A},\hat{u},\hat{p}$ of the *j*-ANN are non-dimensionalized with the following transformations:

(A2) $${\hat{u}}^{*}=\frac{\hat{u}}{U},\;{\hat{A}}^{*}=\frac{\hat{A}}{A^{0}},\;{\hat{p}}^{*}=\frac{p}{p_{0}},\;x^{*}=\frac{x}{L},\;t^{*}=\frac{t}{T},$$

where $p_{0}=\rho U^{2}$, $T=\frac{L}{U}$, and $A^{0}=L^{2}$. This transformation is also performed for the boundary conditions as $u^{*}=\frac{u}{U}$ and $A^{*}=\frac{A}{A^{0}}$ in the loss function (9). The above transformation ensures that $\left({\hat{A}}^{*},{\hat{u}}^{*},{\hat{p}}^{*},x^{*},t^{*}\right)∼O\left(1\right)$. Furthermore, it is well known that normalizing the neural network inputs—in this case, the spatial and temporal variables—mitigates gradient vanishing problems [26]. Applying normalization in vessel *j*, we define

(A3) $${\hat{x}}^{*}=\frac{{x^{*}}^{j}-\mu_{x^{*}}^{j}}{\sigma_{x^{*}}^{j}},\;{\hat{t}}^{*}=\frac{{t^{*}}^{j}-\mu_{t^{*}}^{j}}{\sigma_{t^{*}}^{j}},$$

where $\mu_{x_{*}}^{j},\mu_{t_{*}}^{j}$ are the means, and $\sigma_{x_{*}}^{j},\sigma_{t_{*}}^{j}$ are the standard deviations of the spatial and temporal coordinates of vessel *j*, respectively. The dimensionless variables are recovered by multiplying the network predictions by the scaled parameters, i.e., $p=\hat{p}p_{0}$, $u=\hat{u}U$, $A=\hat{A}A^{0}$.

## Appendix B. Validation of the Model

Before clinical data implementation, the proposed method was validated using a publicly available synthetic dataset generated through simulations based on the Discontinuous Galerkin (DG) method by Kissas et al. [13] (https://github.com/PredictiveIntelligenceLab/1DBloodFlowPINNs) (accessed on 14 August 2025). This dataset serves as the reference solution and includes information on area, velocity, and blood pressure in a Y-shaped arterial bifurcation. The parameters used in the simulation are detailed in Table A1.

In the Galerkin-based simulation, the blood parameters used by Kissas et al. were α=1, ρ=1060, and $K_{R}=0$ for (1) to (5). The external pressure in the three vessels was set to zero. As initial conditions, the velocity was set to zero in each of the three blood vessels, while the initial area was defined according to the data in Table A1. Subsequently, time series of velocity and area were extracted from this reference solution at the initial and final points of the arterial bifurcation. These time series were used as input data for the loss function component associated with clinical measurements, following the scheme presented in Section 2.2.

**Table A1:** Physiological parameters used in the simulation with the DG method.

Artery Segment	Length (m)	β (Pa/m)	Cross-Sectional Area at Equilibrium (m^2^)
1	0.1703	$6.97\times 10^{7}$	$1.36\times 10^{-5}$
2	0.007	$5.42\times 10^{8}$	$1.81\times 10^{-6}$
3	0.0067	$6.96\times 10^{7}$	$1.36\times 10^{-5}$

Figure A1A shows the evolution of the loss function during training for the first 90,000 iterations, using a learning rate of 0.001 with the Adam optimizer. Figure A1B, on the right, illustrates how the loss continued to decrease after 40,000 additional iterations, with a lower learning rate (0.0001), using the same optimizer.

Finally, the observed comparisons confirm mass and momentum conservation in the implemented model. Specifically, the flow in vessel 1 is equal to the sum of the flows in vessels 2 and 3 at the bifurcation point. Additionally, the momentum in all three blood vessels aligns correctly at the bifurcation point, validating the proposed model’s ability to preserve fundamental conservation laws.

**Table A2:** Mean squared error corresponding to each vessel. The first row shows the error between the velocity $u^{j}$ predicted by the network and the reference velocity $u_{ref}$. The second row presents the error between the pressure $p^{j}$ predicted by the network and the reference pressure $p_{ref}$. The third column corresponds to the relative error for pressure.

	Artery 1	Artery 2	Artery 3
$∥u_{ref}-u^{j}{∥}_{L_{2}}$	0.0937282[m/s]	0.02488884[m/s]	0.028534304[m/s]
$∥p_{ref}-p^{j}{∥}_{L_{2}}$	1496.4905[Pa]	1377.7694[Pa]	496.27707[Pa]
$\frac{∥p_{ref}-p^{j}{∥}_{L_{2}}}{∥p_{ref}{∥}_{L_{2}}}$	0.029788	0.029793	0.009996
